# Improving the therapeutic efficacy of oncolytic viruses for cancer: targeting macrophages

**DOI:** 10.1186/s12967-023-04709-z

**Published:** 2023-11-22

**Authors:** Zhen Shen, Xiyu Liu, Guixiang Fan, Jintong Na, Qiaoqiao Liu, Faquan Lin, Zhikun Zhang, Liping Zhong

**Affiliations:** 1 State Key Laboratory of Targeting Oncology, National Center for International Research of Bio-Targeting Theranostics, Guangxi Key Laboratory of Bio-Targeting Theranostics, Collaborative Innovation Center for Targeting Tumor Diagnosis and Therapy, Guangxi Medical University, Nanning, 530021 China; 2 Department of Clinical Laboratory, the First Affiliated Hospital of Guangxi Medical University, Nanning, 530021 Guangxi China; 3 Pharmaceutical College, Guangxi Medical University, Nanning, 530021 Guangxi China

**Keywords:** Cancer immunotherapy, Oncolytic virus, Virotherapy, Macrophage, Macrophage reprogramming, Innate immunity

## Abstract

Oncolytic viruses (OVs) for cancer treatment are in a rapid stage of development, and the direct tumor lysis and activation of a comprehensive host immune response are irreplaceable advantages of cancer immunotherapy. However, excessive antiviral immune responses also restrict the spread of OVs in vivo and the infection of tumor cells. Macrophages are functionally diverse innate immune cells that phagocytose tumor cells and present antigens to activate the immune response, while also limiting the delivery of OVs to tumors. Studies have shown that the functional propensity of macrophages between OVs and tumor cells affects the overall therapeutic effect of oncolytic virotherapy. How to effectively avoid the restrictive effect of macrophages on OVs and reshape the function of tumor-associated macrophages in oncolytic virotherapy is an important challenge we are now facing. Here, we review and summarize the complex dual role of macrophages in oncolytic virotherapy, highlighting how the functional characteristics of macrophage plasticity can be utilized to cooperate with OVs to enhance anti-tumor effects, as well as highlighting the importance of designing and optimizing delivery modalities for OVs in the future.

## Introduction

Tumors often acquire immunosuppressive mechanisms through cancer immunoediting, which shapes the tumor microenvironment (TME) for tumor growth, effectively avoiding immune-mediated tumor clearance [1, 2]. In fact, cells in the TME are both tumor-supportive and tumor-suppressive, and the function of these cells is influenced by the type of cancer, the individual development of the TME cells, and the degree of their “education” [3].

Currently, tumor immunotherapy has received increasing attention with the focus on reducing tumor-associated immune evasion as the main therapeutic strategy for cancer treatment and depleting or “re-educating” cancer-promoting microenvironment cells into an immune-stimulating phenotype [3, 4], which serves as an effective means of cancer treatment by interfering with the body's immune response, and has triggered a revolution in the field of oncology [5, 6].

Oncolytic virotherapy is an emerging immunotherapy, where oncolytic viruses (OVs) preferentially infect tumor cells to suppress tumor growth and are safe for other normal cells. Above this, OVs can activate the host immune system to generate an immune response to enhance anti-tumor efficacy [7], which can overcome cancer-associated immunosuppression and reshape the tumor microenvironment by increasing the infiltration of immune effector cells in “cold” tumors, making them “hot”. This immune response is becoming a decisive factor in the efficacy of viral therapy [8, 9].

However, since OVs are essentially immunogenic infection factors, they stimulate the host to generate an immune response that can both produce antitumor effects and limit the activity of the virus itself [10]. The delivery of OVs to the host initiates a series of immune responses, and macrophages are one of the professional antigen-presenting cells that bridge innate and adaptive immunity, with major roles in both anti-pathogen infection and anti-tumor immunity [11, 12]. However, the clearance of tumor cells and viruses by macrophages is complex, which is determined by the phenotype and function of macrophages. For one, phagocytosis and antigen presentation by macrophages synergize with OVs to inhibit tumor growth, while OVs also stimulate the shift of macrophages to an antitumor phenotype; as for another, macrophages in turn mediate the clearance of OVs through the secretion of interferon type I (IFN) and the phagocytosis of OVs particles, a role that is an important factor in limiting the systemic administration of OVs [13, 14].

The functional tendency of macrophages between OVs and tumor cells affects the overall therapeutic effect of oncolytic virotherapy, and how to eliminate the detrimental effect of macrophages on OVs and make them work in an anti-tumor direction is an important challenge nowadays. Therefore, in this review, we discuss the essential features and interactions between OVs and macrophages in antitumor immunity, and explore strategies by which to intervene with macrophages in oncolytic virotherapy to improve efficacy.

### Oncolytic virotherapy and antitumor immunity

**Fig. 1:**
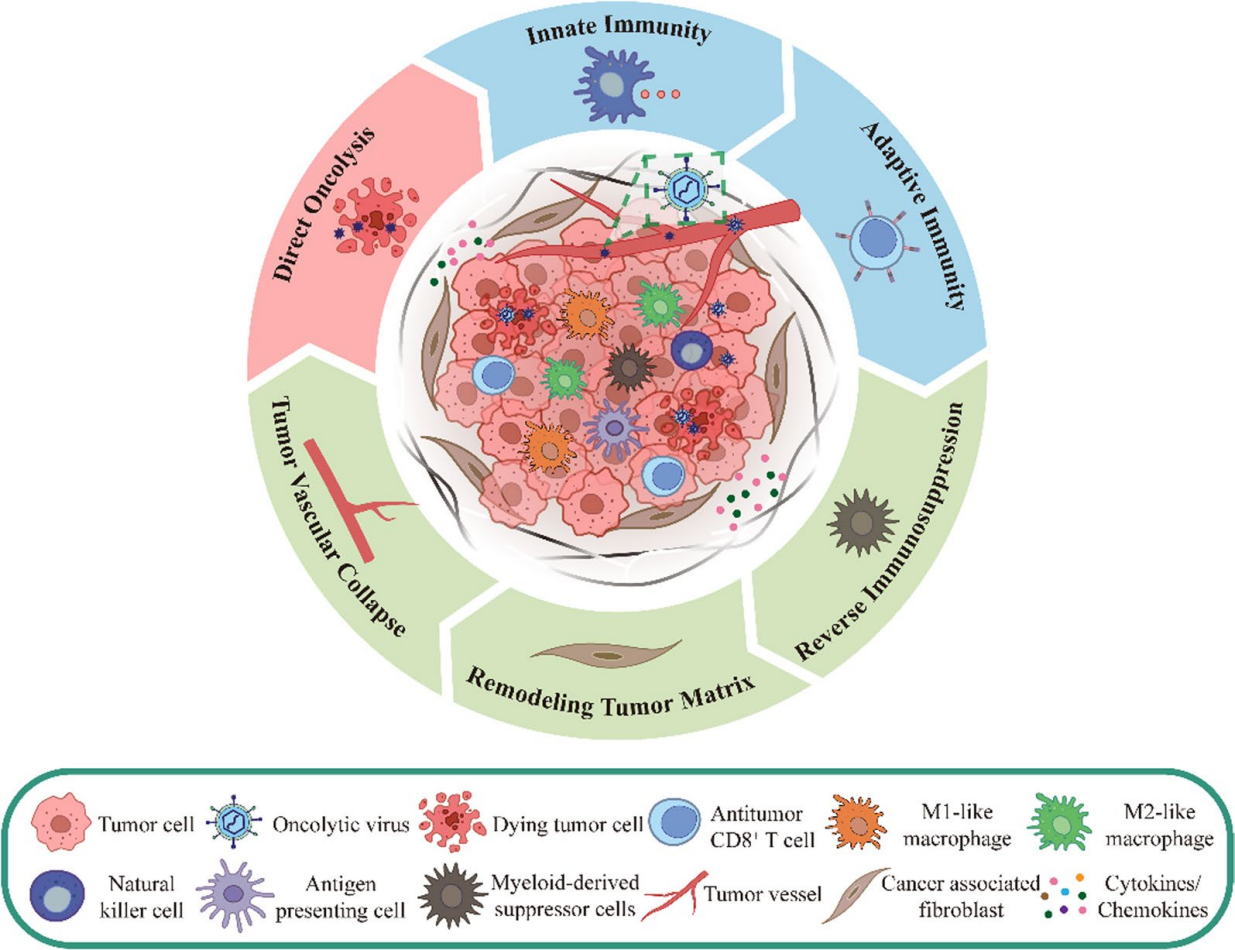
Basic anti-tumor mechanisms of OVs. OVs inhibit tumor growth by selectively infecting and lysing tumor cells, inducing anti-tumor innate and adaptive immunity in the body, and modulating immunosuppressive cells, extracellular matrix cells, and inhibiting tumor angiogenesis in the TME

Compared with conventional tumor immunotherapy, oncolytic virotherapy has several advantages, including significant killing effect, high degree of targeting, low adverse effects, and less susceptibility to drug resistance [15, 16]. This is related to the unique antitumor mechanism of OVs, which induces tumor death based on natural interactions among viruses, tumor cells and TME, and the body's immune system **(**Fig. 1**)**, including direct lysis of tumor cells, promotion of immunogenic cell death (ICD), induction of innate and adaptive immune responses, regulation of TME, and inhibition of tumor angiogenesis [17–19]. Furthermore, OVs can be genetically engineered to exert specific effects to enhance anti-tumor functions, the most prevalent of which include tumor targeting, expression of pro-inflammatory cytokines, and tumor suppressor genes [20, 21].

### Direct tumor lysis

In normal host cells, OVs are sensed and cleared by activated antiviral signaling pathways, thus protecting normal cells from OVs damage. However, in tumor cells, overexpression of tumor antigens favorable for OVs binding, availability of a large number of nucleotides, aberrant activation of the oncogenic pathway, dysregulation of the apoptotic pathway, and deficiency of antiviral type I IFN signaling provide OVs with a suitable growth environment and a shelter [22, 23], which allows them to selectively infect and destroy tumor cells, and further releases more progeny of OVs for spreading to neighboring uninfected tumor cells, resulting in an increasing lysis effect [15].

### Enhance host immune response

Another important role of OVs is the enhancement of the antitumor immune response through the body-activated antiviral immune response. After delivery of OVs, the immune system preferentially initiates an antiviral response, including the release of antiviral cytokines such as IFNs, tumor necrosis factor-α (TNF-α), and interleukin 12 (IL-12) [24], with these cytokines promoting the maturation of dendritic cells (DCs), which induces a natural antiviral immune response in macrophages and natural killer (NK) cells. Activated NK cells expressing γ-interferon (IFN-γ) and TNF-α contribute further to the activation of macrophages, DCs, and T cells, amplifying the immune response [8, 20], which are recruited to the site of virus-infected tumors, and altering the cytokine environment and the immune cells in the TME, thereby overcoming tumor-associated immunosuppression [25]. Tumor cells invaded and lysed by OVs release progeny viruses, pathogen-associated molecular patterns (PAMPs), damage-associated molecular patterns (DAMPs), tumor-associated antigens (TAAs), and neoantigens to the extracellular space, and the resulting progeny viruses spread to the surrounding area and infect more tumor cells, creating a tendency for tumor lysis. Whereas DAMPs and PAMPs stimulate the immune system by activating pattern recognition receptors (PRRs) such as Toll-like receptors (TLRs) on a variety of immune cells, initiating cellular activation and inflammatory signaling [9]. TAAs and neoantigens are taken up by antigen-presenting cells (APCs) and delivered to T cells, generating viral/tumor antigen-specific CD8^+^ T cells, which create an immunostimulatory microenvironment at the tumor site, while at the same time, these variations contribute to the transformation of tumor-supportive M2-like macrophages into tumor-suppressive M1-like macrophages [24].

### Effect of OVs on TME

The TME provides favorable conditions for tumorigenesis and progression. TME usually includes infiltrative inflammatory cells such as macrophages, lymphocytes, NK cells, and DCs; immunosuppressive cells such as myeloid-derived suppressor cells (MDSCs), regulatory T cells (Tregs), and tumor-associated macrophages (TAMs); cancer-associated fibroblasts (CAFs) tumor stromal cells, and tumor vascular system [3, 26]. Tumor cells co-secrete growth factors, cytokines, and chemokines with immunosuppressive cellular cells that inhibit the normal anti-tumor immune response, provide support for tumor development and angiogenesis, promote tumor progression, and limit therapeutic response [26–29].

Natural or modified OVs can remodel the immunosuppressive TME, increase the influx of anti-tumor immune cells such as macrophages, NK cells, DCs, T cells, and neutrophils, convert MDSCs into a tumor-killing phenotype, reduce the populations of immune-suppressing cells, remodel the tumor extracellular matrix, and resist tumor angiogenesis, thus promoting tumor elimination by making immunologically “cold” tumors “hot” [30, 31].

In short, OVs can act directly or indirectly on different parts of the tumor ecosystem such as tumor cells, immune cells and cytokines, tumor stromal cells, and tumor vasculature system to inhibit tumor progression.

## Macrophages and antitumor immunity

### Origin and distribution of macrophages

Macrophages are ubiquitous in any part of the body and perform three essential functions, namely phagocytosis, exogenous antigen presentation, and secretion of cytokines and growth factors for immunomodulation. They perform important duties in tissue development, homeostasis, clearance of dead cells and foreign pathogens, and modulation of inflammatory and tumoral immune responses [32–34]. Macrophages also have different names and functions in different tissues, such as circulating monocyte-derived macrophages, tissue-resident macrophages (TRMs), and tumor-associated macrophages, which have complex correlations in terms of classification and origin. TRMs perform appropriate functions in various tissues of the body, including microglia in the brain, Kupffer cells in the liver, and Langerhans cells in the skin [35, 36], and it is currently believed that most of the population of TRMs originates from embryonic precursors in the yolk sac and fetal liver and that they self-maintain independently of the myeloid cells in adulthood [37, 38]. TAMs, on the other hand, consist mainly of circulating monocyte-derived macrophages and RTMs recruited by tumors into TME and are one of the important targets for tumor immunotherapy [39].

### Phenotype and function of macrophages

**Fig. 2:**
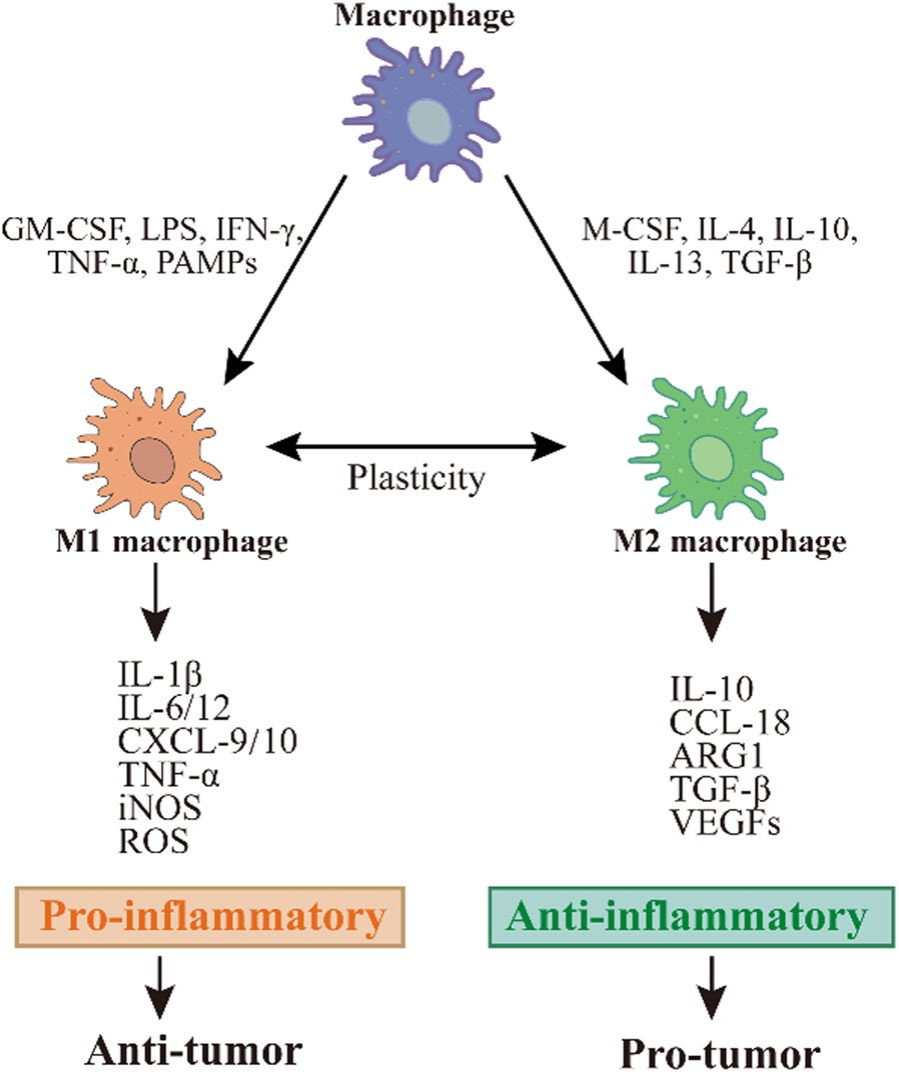
Macrophage activation and M1/M2 typing. Macrophages polarized into classically activated (M1) or alternatively activated (M2) macrophages under the influence of different cytokines or other factors secrete different cytokines to change the cellular microenvironment to a pro-inflammatory or anti-inflammatory state, exerting anti-tumor or pro-tumor effects at the tumor site

Macrophages are significant plastic and their activation state is influenced by a multitude of factors, but they can usually be simplified into two classifications based on stimulatory factors and secretory products **(**Fig. 2**)**, namely classically activated M1 macrophages and alternatively activated M2 macrophages [40]. Although this M1/M2 dichotomization simplifies the differences in phenotypic and functional continuum changes in macrophages, this terminology is still more commonly used when discussing whether macrophages are more biased toward a pro-inflammatory or anti-inflammatory phenotype [41].

M1 macrophage polarization is usually driven by granulocyte–macrophage colony-stimulating factor (GM-CSF), lipopolysaccharide (LPS), IFN-γ, TNF-α, and PAMPs [42]. M1 phenotype macrophages have mainly pro-inflammatory properties, promoting the pro-inflammatory response of helper T cells 1 (Th1) by secreting cytokines, such as TNF-α, IL-1β, IL-12, and IL-18, and enhancing the recruitment of Th1 cells to sites of inflammation by secreting chemokines, such as chemokines CXC motif ligand 9 (CXCL9) and CXCL10 [43]. M1 macrophages can trigger an adaptive immune response through self-mediated cytotoxicity or cross-presentation of antigens (TAAs and TANs), triggering potent anti-tumor immunity. Therefore, M1 macrophages are considered a tumor-suppressive macrophage phenotype [44].

M2 macrophage polarization is usually driven by macrophage colony-stimulating factor (M-CSF), IL-4, IL-10, IL-13, and transforming growth factor-β (TGF-β) [45]. M2 macrophages have a critical position in appropriate immune function and homeostasis in vivo, with examples including stimulation of Th2 cell responses, mediation of parasite clearance, immunomodulation, wound healing and tissue repair [46]. However, the function of M2 macrophages can also be adversely affected by tumor exploitation by producing immunosuppressive and pro-angiogenic factors such as IL-10, arginase 1 (ARG1), TGF-β, or vascular endothelial growth factors (VEGFs), which stimulate tumor cell proliferation, invasion, metastasis, and angiogenesis [41]. Therefore, M2 macrophages are considered a tumor-supporting macrophage phenotype[47].

### Tumor-associated macrophages (TAMs)

TAMs are a collective term for macrophages that are prevalent in tumors and can account for up to 50% of some solid tumors [48]. TAMs also share the markers of M1/M2 macrophages [49], however, TAMs rarely exhibit a true M1 or M2 phenotype and are more aptly referred to as M1-like/M2-like TAMs [50]. Under the effects of tumor-secreted colony-stimulating factor 1 (CSF-1, or M-CSF), TAMs polarize to M2-like, allowing immunosuppressive M2-like TAMs to predominate in tumors [47, 51]. High infiltration of M2-like TAMs reduces therapeutic efficacy, shaping tumor-supportive TME, angiogenesis, fibrosis, immunosuppressive cell recruitment, lymphocyte rejection, drug resistance, invasion, and metastasis to enhance tumor progression [52–54], which are often associated with poor clinical outcomes [55–57].

TAMs are effective target cells in immunotherapy of tumors [12, 58]. This is because macrophages exert opposite anti-tumor or pro-tumor functions through a range of activation pathways and/or different macrophage populations [13, 59]. Different approaches can be taken to eliminate tumor-promoting macrophages and activate or transform them into tumor-suppressing macrophages. Common therapeutic strategies are inhibition of TAMs recruitment [60, 61], reprogramming of TAMs to an M1-like phenotype [62–64], and depletion of TAMs [65, 66].

### Interaction of OVs, macrophages, and tumors

**Fig. 3:**
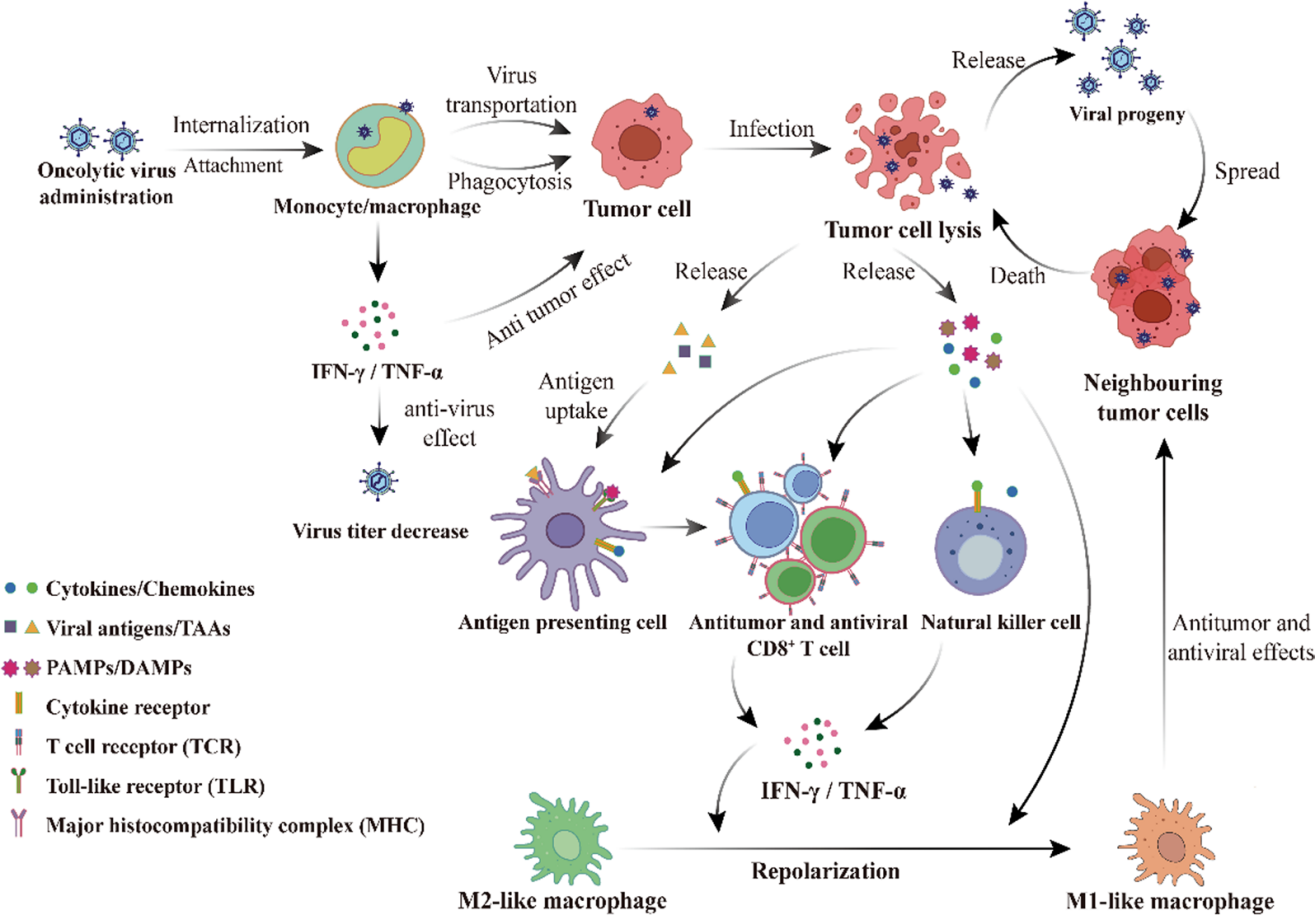
Interaction of OVs, macrophages, and tumor cells. After OVs are delivered, some OVs are attacked by activated monocytes/macrophages, causing the viral titer of OVs to decrease. Another portion of OVs can be transported to the tumor site for viral replication, lysing tumor cells and releasing viral progeny, damage-associated molecular patterns (DAMPs), pathogen-associated molecular patterns (PAMPs), and tumor-associated antigens (TAAs). Antigen-presenting cells (APCs) take up and present these antigens, and the resulting activated antigen-specific CD8 + T cells as well as natural killer (NK) cells exert antitumor effects. Secreted IFN-γ and PAMPs repolarize pro-tumorigenic M2-like macrophages into anti-tumorigenic M1-like macrophages, and the anti-tumor/viral effects of the immune system can be further enhanced by secreting IFN-γ and TNF-α

Macrophage plasticity influences tumor progression and treatment outcome and has a similar effect in oncolytic virotherapy. When OVs are delivered to the body, the body triggers innate immunity in response to the “foreign invasion” of viral infection. Monocytes, macrophages and NK cells will recognize and remove some of the OVs and play a certain inhibitory role. However, in this process, macrophages will also act as carriers of OVs to tumor cells. At the same time macrophages enhance polarization toward a pro-inflammatory phenotype, and this local immune response is also critical for initiating initial anti-tumor immunity [67]. Therefore, we need to further comprehend the complex interactions among OVs, macrophages, and tumors **(**Fig. 3**)**, to elucidate the mechanisms of macrophages that limit or promote the tumoricidal effects of OVs, and to better utilize the advantages of macrophages to enhance the anti-tumor benefits in future oncolytic virus therapeutic strategies.

### Macrophages limit the antitumor effect of OVs

In general, macrophages show antiviral activity in the setting of oncolytic virotherapy, which is consistent with their defense against pathogens.

Among the routes of administration of OVs, intravenous has more potential than intra-tumoral injection in the treatment of systemic metastatic tumors. However, intravenously administered OVs are often hindered by circulating and tissue immune complexes, neutralizing antibodies, and innate immune cells before reaching the tumor site. Activated macrophages have multiple viral clearance mechanisms, including virus recognition through PRRs, cytokine responses such as IFN, phagocytosis, and activation of other immune cells to reduce viral titers delivered to the tumor site [68, 69].

In a glioma model, phagocytosis by macrophages limits the spread of OVs. Delivery of oncolytic herpes simplex virus (oHSV) after the depletion of macrophages can increase viral titers at tumor sites [70]. IFN and TNF-α signaling is an important mechanism for the antiviral effects of macrophages [71, 72]. In ovarian and breast cancer models it was shown that macrophages can activate the tumor cell JAK/STAT pathway and upregulate the expression of interferon-stimulated genes (ISGs), with tumor cells thereby acquiring an antiviral status that makes them resistant to OVs [73]. In a study of glioblastoma (GBM) treated with oHSV, macrophages, and microglia were found to be the main producers of TNF-α, which inhibits viral replication. Brief administration of TNF-α blockers effectively enhances the killing of tumor cells while reducing inflammation-induced neurotoxicity, enhancing viral replication and survival in GBM intracranial tumors [69]. TAMs and microglia in malignant gliomas largely limit the activity of OVs [74].

Although inflammatory cytokines and phagocytosis produced by macrophages are powerful weapons to kill tumor cells, they also reduce the efficiency of transport of OVs to tumors, so direct delivery of OVs requires a larger viral load to counteract this clearance effect and increases the viral titer of transport to tumor sites.

### Macrophages promote the antitumor effect of OVs

However, on the other hand, the interaction between macrophages and OVs could enhance the antitumor effect.

First of all, macrophages can act as carriers of OVs for transport. Macrophages have shown antiviral effects to some extent, but interestingly, increasing studies have evidenced that viruses can utilize monocytes/macrophages as vectors for spreading and replication [75], and macrophages may be an integral part of the therapy of OVs, possibly due to the higher susceptibility of monocytes or naïve macrophages to OVs [76]. Previous research has found that monocytes/macrophages in peripheral blood can act as viral vectors, transporting viable viral particles to tumor sites. Follow-up after intravenous administration of the eutherian virus recovered replicative and oncolytic eutherian virus in blood mononuclear cells even in the presence of neutralizing antibodies (nAbs) to the virus [77]. In another study with oncolytic adenovirus, it was shown that, possibly due to the very low expression of viral antigens, macrophages can act as silent vectors that hide and support viral replication, allowing adenovirus delivery to the tumor site and produce a long-lasting therapeutic effect [78]. More interestingly, recent preclinical studies have found that macrophages are not only capable of uptake and delivery of the tumor oncolytic virus HSV1716 but also support HSV1716 replication within macrophages, which could enhance the effect of viral therapy [79].

Second, OVs can enhance the phagocytic activity of macrophages on tumor cells. As mentioned earlier, TAMs are an important component of macrophages. Activation of TAMs to produce phagocytic activity is a novel mechanism of tumor killing [80], which can be activated by oncolytic virus treatment. CD47 is a membrane-bound protein that is highly expressed on tumor cells and binds to signal regulatory protein α (SIRPα) on macrophages, delivering a “don't eat me” signal that leads to immune evasion by the tumor [81]. After OVs infect cells, PAMPs are exposed to the host immune system, inducing endoplasmic reticulum stress and ICD, leading to the release of DAMPs [82–84], which include calreticulin (CRT). CRT, an endoplasmic reticulum-associated molecular chaperone, can also block the CD47 receptor on tumor cells, thereby reducing the “don't eat me” signals generated by macrophages and DCs in response to CD47 binding, and attenuating immune evasion by tumor cells [85]. In addition, after OVs interacted with the B cell receptor (BCR), activated B cells were able to release neutralizing antibodies that mediated NK cell antibody-dependent cytotoxicity (ADCC) and macrophage antibody-dependent cell phagocytosis (ADCP) of virus-infected tumor cells, activating phagocytosis of tumor cells by innate immune cells [86].

Most importantly, OVs can induce polarization of TAMs towards an anti-tumor phenotype. OVs induce activation of NK cells and macrophages through PRRs recognizing PAMPs and DAMPs, secretion of inflammatory cytokines such as IFN-γ, and induced macrophage polarization to M1-like, which results in diminished immunosuppression of TAMs [76, 87]. In an in vitro model of breast cancer, it was found that irrespective of the initial polarization state of macrophages, treatment with oncolytic measles virus (MeV) and mumps virus (MuV) resulted in a significant increase in the M1 macrophage marker, CD80, in human monocyte-derived macrophages (MDMs), while inducing anti-tumor cytokines IL-1β, TNF-α, CXCL9, CXCL10, and IL -6 concentrations were elevated [88]. Preclinical and clinical studies in gastric cancer or glioma have found that treatment with HSV-1 or oncolytic adenovirus rapidly recruited inflammatory cells to the injected lesions, significantly increased the intra-tumoral infiltration of M1-like macrophages and NK cells, with a reduction in the expression of M2-like macrophages, and a significant elevation of the pro-inflammatory cytokines IFN-γ and TNF-α [89, 90]. Although oncolytic adenovirus shifts human macrophages from a more pro-tumor phenotype to a less favorable phenotype, this phenotypic shift is not complete and the M2 trait is not completely lost at the level of gene expression, immunophenotype, and cytokines, which is consistent with the concept that the M1/M2 typing of macrophages is not completely extreme, but rather sequential in phenotype and function [91].

### Enhance the synergistic anti-tumor effect of OVs and macrophages

Due to the multifaceted effects generated by macrophages in the treatment of OVs, eliminating the limiting effect of macrophages on OVs, exploiting the effectiveness of macrophages, and obtaining better therapeutic results require intensive research. The current directions are mainly the following: (1), arming OVs to enhance the beneficial effects (pro-inflammatory phenotypic polarization and phagocytosis) or attenuate the adverse effects (antiviral and pro-tumorigenic effects); (2), combining with other drugs to increase the antitumor efficacy; and (3), augmenting the targeting of OVs to tumor cells through effective carrier delivery.

### Arming of OVs

**Fig. 4:**
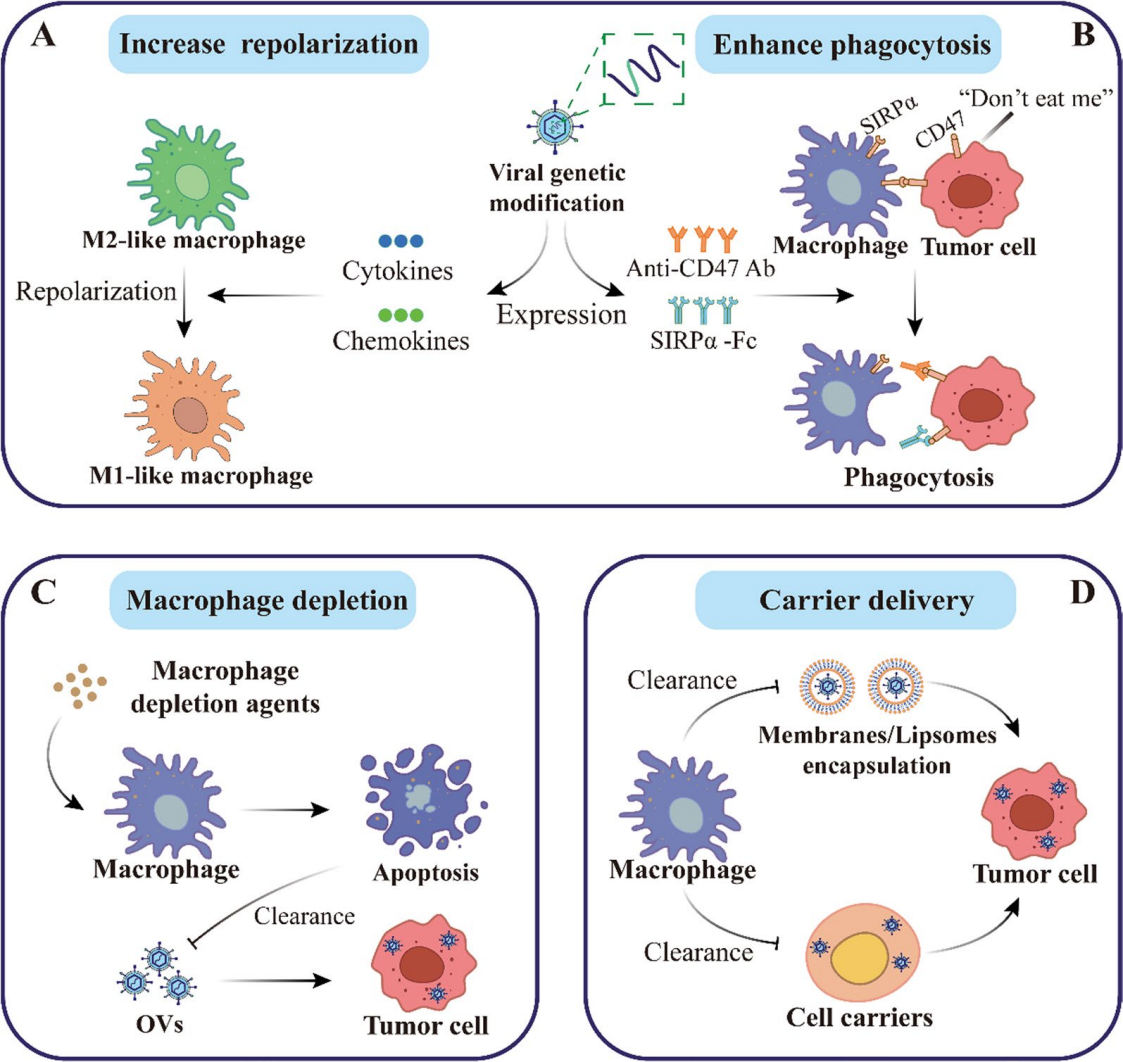
Basic macrophage strategies in oncolytic virotherapy. Currently, there are two major directions of basic strategies for targeting the macrophage to optimize therapeutic response. On the one hand, armed OVs enhance the anti-tumor effect of macrophages. **A** Repolarization to an antitumor phenotype. Given the pro-tumorigenic role of M2-like tumor-associated macrophages (TAMs), the expression of pro-inflammatory cytokines or chemokines by genetically modified viruses was used to increase macrophage activity and promote the polarization of M2-like macrophages to M1-like macrophages. **B** Enhancement of phagocytosis by macrophages. The expression of anti-CD47 antibody or SIRPα-Fc fusion protein after viral genetic modification can disrupt “don't eat me” signaling and enhance the killing of tumor cells by macrophages. On the other hand, weakening the clearance of OVs by macrophages contributes to higher viral titers at tumor sites. **C** Direct macrophage depletion. Since OVs are subject to phagocytosis by macrophages and/or clearance by antiviral cytokines after delivery, brief administration of macrophage depletion agents prior to OVs treatment can cause apoptosis of macrophages, increase the titer of OVs, and change the phenotype of TAMs. **D** Delivered through the carrier. In addition, the use of tumorophilic carrier cells or liposomes to deliver OVs, is also able to avoid the negative effects of neutralizing antibodies and/or innate immune cells and overcome the challenges of systemic administration of OVs

OVs can be genetically engineered to arm viruses, and different immunomodulatory genes for arming OVs are being actively tested. Various OVs expressing pro-inflammatory cytokines, chemokines, and other immune checkpoint-associated molecules have been developed to enhance the anti-tumor effects of macrophages **(**Fig. 4A**)**.

#### Arming of OVs to enhance macrophage repolarization

A high M2/M1 ratio in TAMs is strongly associated with tumor progression and poor prognosis. Although OVs can inherently promote polarization of M1-like TAMs and reduce the number of M2-like TAMs, armed OVs can further enhance this polarization.

Talimogene laherparepvec (T-VEC), a GM-CSF-expressing HSV-1, is the first OVs approved by the U.S. Food and Drug Administration (FDA) for the treatment of patients with advanced melanoma, with favorable safety and therapeutic outcomes [92]. This is due to the ability of GM-CSF-expressing OVs to attract monocytes and differentiate them into macrophages and DCs, repolarize TAMs from an M2-like phenotype to an M1-like phenotype, and increase the expression of the pro-inflammatory cytokines TNF-α, IL-6, and IL-10 [93, 94].

IL-12 is one of the major regulators of anti-tumor immune responses, promoting the maturation of NK cells, DCs, and T cells, inducing M1-like polarization of macrophages, and increasing IFN-γ levels [95]. Many OVs are currently modified and produce IL-12 [96], and in a GBM model, the use of an oHSV expressing murine IL-12 (G47Δ-mIL12) increased polarization of M1-like TAMs (iNOS^+^ and pSTAT1^+^), which may be due to IL-12-induced increases in IFN-γ in the TME [97].

Although IL-12 can effectively induce antitumor immunity, it has certain toxic side effects after systemic administration [95], and IL-21 may be a safer cytokine compared to IL-12. In a pancreatic cancer model study, it was demonstrated that treatment with VVL-21, an oncolytic vaccinia virus (VV) that expresses IL-21, increased the expression of M1-like macrophage marker major histocompatibility complex II (MHC II) and cytokine gene transcripts (IL-6/IL-12 and COX2), and decreased the expression of M2 macrophage marker (CD206) and cytokine gene transcripts (IL-10, TGF-β, and CCL22) expression while also increasing M1 polarization in naïve macrophages [98]. In addition, an IL-36γ-expressing VV (IL-36γ-OVs) was developed. It induces infiltration of lymphocytes and DCs, reduces MDSCs and M2-like TAMs, and has shown significant therapeutic effects in a variety of mouse tumor models [99].

OVs with chemokines are able to effectively recruit immune cells with antitumor effects to migrate to infected tumor sites. Chemokine CC motif ligand 5 (CCL5) promotes immune cell chemotaxis by interacting with chemokine CC motif receptor 1 (CCR1), CCR3, and CCR5 [100]. Infection of tumor cells with CCL5-expressing OVs significantly enhances the migration and activation of NK cells, macrophages, and T cells, and also activates the secretion of CXCL9 by macrophages and DCs aggregated in tumors by binding to tumor cells to activate Fc receptor-mediated ADCC in NK cells and ADCP in macrophages [101, 102], which in turn further promotes the infiltration of circulating T cells into tumor tissues [103].

Both CD40 and OX40 and their ligands CD40L and OX40L belong to the TNF receptor superfamily (TNFRSF). The interaction of CD40 and CD40L activates APCs [104], and the interaction of OX40 and OX40L activates T cells [105], which promotes antitumor effects through activated downstream signaling pathways. A CD40L-expressing oncolytic adenovirus (TMZ-CD40L) is effective in treating pancreatic cancer, a tumor with a high level of M2 macrophages, by increasing the infiltration of M1-like macrophages and T cells into the tumor, repolarizing M2-like macrophages, and controlling tumor progression [106]. Also in a pancreatic cancer model, the use of HSV-1 expressing murine OX40L ((OV-mOX40L) triggered an OX40-OX40L signaling pathway-mediated response that also reprogrammed macrophages and neutrophils to an anti-tumor state, enhanced the anti-tumor response of T cells, and significantly prolonged the survival time of mice [107].

#### Arming of OVs to enhance phagocytosis

At the same time, it is desired to modify OVs to further block the immunosuppressive effect and enhance phagocytosis of tumors by macrophages **(**Fig. 4B**)**. An engineered oHSV equipped with a full-length anti-CD47 antibody can be used to disrupt the “don't eat me” signaling generated by the CD47/SIRPα pathway. This oHSV activated phagocytosis and cytotoxicity of tumor cells by macrophages and NK cells, prolonging the survival of glioblastoma and ovarian cancer model mice [108, 109]. Accordingly, investigators designed a VV capable of expressing a chimeric molecule (SIRPα-Fc) consisting of the ectodomain of SIRPα and the Fc structural domain of IgG4. SIRPα-Fc was able to disrupt CD47/SIRPα interactions by blocking CD47 in tumor cells, redirecting macrophages to the tumor site and killing the tumor cells. This VV exerted potent anti-tumor activity in a mouse model of osteosarcoma and can be broadly applied to tumors expressing CD47 [110].

Recently, in a study on cholesterol metabolism, progress has also been made in relation to macrophage phagocytic activity. This study found that TAMs in GBM accumulate cholesterol abnormally, leading to dysfunctional phagocytosis [111]. Apolipoprotein A1 (ApoA1) is a cholesterol reverse transporter protein that allows cholesterol efflux from TAMs, thereby restoring their phagocytosis and antigen-presenting role. Therefore, the investigators developed an ApoA1-expressing oncolytic adenovirus (AdV^APOA1^) to intervene in cholesterol metabolism in GBM. AdV^APOA1^ activated the TAM-T cell axis and downregulated immune checkpoints after intra-tumor administration, inducing systemic tumor-specific immune memory [111]. This study proposes an immunometabolic treatment approach to armed OVs.

#### Arming OVs to attenuate the adverse effects of macrophages

Genetically modified OVs not only enhance anti-tumor immunity in macrophages, but also circumvent the detrimental effects of macrophages, including reducing M2-like TAMs and attenuating macrophage-restricted effects on OVs.

Currently, a panel of oncolytic adenoviruses (EnAd) expressing bivalent T-cell engagers (BiTEs) has been designed to target the immunosuppressive effects of M2-like TAMs. The BiTEs recognize CD3ε on T cells and CD206 or folate receptor β (FRβ) on M2-like macrophages. Use of such OVs in patients with malignant ascites activates T cells to selectively kill M2-like macrophages, thereby preserving M1-like macrophages and repolarizing the microenvironment toward a pro-inflammatory state [112].

Human species C adenovirus (HAdv-C5) is bound by immunoglobulin M (IgM) and coagulation factor X (FX) in the blood when delivered intravenously [113, 114], leading to the sequestration of OVs in liver-resident macrophages (Kupffer cells), limiting their tumor targeting and leading to hepatotoxicity [115]. Based on these, the investigators constructed the HAdv-C5 capsid-modified viral variant Ad5-3 M. Ad5-3 M is resistant to IgM- and complement-mediated inactivation, reduces internalization of the viral variant by Kupffer cells, and circumvents the adverse effects of innate immunity to OVs. In mice with disseminated lung tumors, Ad5-3 M prolonged survival and improved safety and efficacy after intravenous administration of OVs [116]. Therefore, the use of genetic modification to change some protein sites in OVs to enhance their resistance is also a worthy direction.

### Combination therapy with OVs

In addition to modifying the OVs' own properties, finding the appropriate drugs for combination therapy opens up more possibilities. These strategies include combining immune checkpoint inhibitors to enhance antitumor effects, and combining macrophage depleting agents or immunosuppressive drugs to increase the titer of OVs.

Combination therapy with OVs and immune checkpoint inhibitors (ICIs) is a common combination strategy in clinical trials today **(**Table 1**)**, due to the ability of OVs to increase the sensitivity of tumor cells to ICIs, which has demonstrated a strong therapeutic effect in a wide range of tumor treatments [117–119]. In a GBM model, the use of IL-21-expressing VV (VVDTK-STCDN1L-mIL21) in combination with systemic anti-programmed death receptor 1 (anti-PD1) therapy showed significant induction of M1-like macrophage polarization in the tumor during treatment, along with increased activation of M0 macrophages (MHC II^+^) in the spleen and DCs in the lymph nodes [120]. Similarly, in other GBM and triple-negative breast cancer models, combination treatment of engineered OVs with ICIs such as anti-cytotoxic T-lymphocyte-associated protein 4 (anti-CTLA-4) antibody, anti-PD-1 antibody and anti-programmed cell death ligand 1 (anti-PD-L1) significantly inhibited tumor growth. The results showed an increase in the proportion of M1-like TAMs, CD4^+^ and CD8^+^ cells, and a decrease in the number of immunosuppressive cells such as Tregs. The application of ICIs prevented immune escape from the tumor and overcame the immunosuppressive microenvironment, which is of great significance for the effective eradication of the tumor [97, 121].

**Table 1:** Clinical trials of OVs combination therapy with immune checkpoint inhibitors

	Virus	RoA	Combination	Cancer	Trial No	Phase	Status
Adenovirus	TILT-123	IT	Avelumab	Advanced solid tumors	NCT05222932	I	Recruiting
	H101	Intravesical	Camrelizumab	Bladder cancer	NCT05564897	II	Recruiting
	NG-350A	IV	Pembrolizumab	Epithelial tumors	NCT05165433	I	Recruiting
	TILT-123	IT/IP	Pembrolizumab	Ovarian cancer	NCT05271318	I	Recruiting
Herpes simplex virus	T-VEC	IT	Panitumumab	Squamous cell carcinoma	NCT04163952	I	Active, not recruiting
	HX-008	IT	Anti-PD-1 monoclonal antibody	Melanoma	NCT05068453 NCT05070221	I	Not yet recruiting
	RP2/RP3	IT	Atezolizumab/Atezolizumab	Colorectal carcinoma	NCT05733611	II	Not yet recruiting
	VG161	IT	Nivolumab	Pancreatic cancer	NCT05162118	I/II	Recruiting
Vaccinia virus	MQ710	IT	Pembrolizumab	Solid Tumors	NCT05859074	I	Recruiting
	ASP9801	IT	Pembrolizumab	Advanced/Metastatic solid tumors	NCT03954067	I	Active, not recruiting
	TBio-6517	IT/IV	Pembrolizumab	Advanced solid tumors	NCT04301011	I/IIa	Active, not recruiting
	BT-001	IT	Pembrolizumab	Advanced solid tumors	NCT04725331	I/IIa	Recruiting
Reovirus	PeLareorEp	IV	Avelumab	Breast cancer	NCT04215146	II	Active, not recruiting
Vesicular stomatitis virus	Revottack	IV	Toripalimab	Advanced malignant solid tumor	NCT05644509	I	Not yet recruiting
Chimeric orthopoxvirus	CF33-hNIS	IT/IV	Pembrolizumab	Advanced solid tumor	NCT05346484	I	Recruiting
M1 virus	M1-c6v1	IV	Camrelizumab	Hepatocellular carcinoma	NCT04665362	I	Not yet recruiting

RoA, Route of Administration; IT, Intratumoral; IV, Intravenous; IP, Intraperitoneal

OVs combined with macrophage-depleting agents have been reported to remodel TME. In macrophage-dependent tumors, investigators tested the effectiveness of clodronate liposomes and trabectedin in the oHSV treatment of Ewing's sarcoma [122]. Clodronate liposomes can transiently deplete macrophages throughout the body and have demonstrated their therapeutic potential in applications in a variety of tumors [70, 123]. Trabectedin is a chemotherapeutic agent that depletes monocytes/macrophages, including TAMs, by activating caspase-8-dependent apoptosis through the TRAIL receptor [65]. Both drugs were found to enhance antitumor efficacy after macrophage depletion **(**Fig. 4C**)**. Clodronate liposomes induced antitumor gene expression in TAMs, trabectedin lowered the number of intratumoral MDSCs and M2-like macrophages, and the combination of both drugs with OVs significantly changed the phenotype of TAMs and tended the immune microenvironment to an inflammatory state [122].

Inhibition of macrophage-associated pathways has also shown good efficacy in combination with other immunologic agents. The phosphatidylinositol-3-kinase (PI3K) pathway has an important part in tumor development. PI3K signaling is a key driver of macrophage M2 polarization [124, 125]. PI3Kδ, one of the classes I PI3K isoforms, is hyper-enriched in leukocytes, of which macrophages are included [126]. Some investigators have demonstrated that treatment with PI3Kδ inhibitors prior to intravenous delivery of VV significantly improves VV delivery to tumors and enhances tumor efficacy. This was achieved by interfering with the RhoA/ROCK, AKT, and Rac signaling pathways to inhibit viral attachment to macrophages, independent of viral internalization by macrophages [127]. They combined a PI3Kδ inhibitor (CAL-101), engineered VV, and α-PD1 for the treatment of pancreatic cancer in mice, and the results showed strong synergistic effects, demonstrated the effectiveness of systemic administration, and broke through a major limitation in the treatment of OVs [98]. In addition to this, the use of rapamycin in oncolytic virotherapy has added new possibilities. Rapamycin has immunosuppressive properties and it is able to reduce type I IFN production by inhibiting mammalian target of rapamycin complex 1 (mTORC1) [128], reduce infiltration of CD68^+^ microglia and CD163^+^ macrophages in gliomas, and increase viral replication and therapeutic efficacy within tumors [129].

### Delivery by carrier

Although suppression of the antiviral immune response of macrophages is beneficial in enhancing the therapeutic effect of OV, such immunosuppression may impair the functional balance of macrophages in vivo and diminish the effect of virus-mediated immune stimulation against cancer. Delivery of OVs using carrier cells with tumorophilic properties can effectively avoid the influence of the immune system and reduce the neutralization and clearance of OVs before they reach the tumor **(**Fig. 4D**)**. Therefore, this approach may be a more desirable strategy to improve the pharmacokinetics and biological distribution of OVs and has been extensively studied in carrier cells such as mesenchymal stem cells (MSCs), T cells, myeloid cells, and neural stem cells [130].

Moreover, the use of tumor cell tropism to enhance tumor targeting has also been studied accordingly. Membrane-encapsulated oncolytic adenovirus from cancer cells delivered intravenously was able to effectively avoid the antiviral effects of neutralizing antibodies and the innate immune system. This system increases viral replication and enhances the ability of macrophages and DCs to present tumor antigens, and has shown good efficacy in the treatment of different mouse tumor models [131]. When using VV in hosts with pre-existing antibodies to poxviruses, the transient use of a combination of multiple immunosuppressive drugs and cancer cells as carrier cells significantly improves therapeutic efficacy. Although this approach is achieved by increasing the polarization of immunosuppressive M2-like TAMs, such changes are necessary in the long run [132].

Encapsulation of OVs via liposomes (LPs) is also one of the attractive nano-delivery systems. Encapsulation of oncolytic adenovirus (Ad[I/PPT-E1A]) into liposomes coupled to chemokine CC motif ligand 2 (CCL2), which upon intravenous delivery binds to circulating monocytes expressing chemokine CC motif receptor 2 (CCR2), takes advantage of the aggregation of monocytes to hypoxic tumor vessels to deliver encapsulated OVs targeting tumor sites [133]. This system can avoid recognition and delivery to the tumor site by the immune system after intravenous delivery, reducing the number of TAMs located near the blood vessels [134].

Therefore, the use of carriers for adjuvant delivery of OVs is one of the promising strategies. This approach evades the capture of OVs by innate immune cells without affecting the body's immune function, while enhancing the targeting of tumors and reducing the viral delivery load.

In conclusion, macrophages are an important factor affecting the therapeutic effect of OVs, and in the face of this dual effect, how to seek benefits and avoid harm is something we need to consider.

## Conclusion and prospect

In conclusion, the wide systemic distribution, plasticity and complex functions of macrophages make them largely influence the outcome and prognosis during oncolytic virotherapy. In the process of tumor development, M1 phenotype macrophages have anti-tumor effects and M2 phenotype macrophages have pro-tumor effects; in oncolytic virotherapy, macrophages can both enhance the effect of viral therapy and limit the spread of OVs to a certain extent. These different regulatory effects may be related to tumor heterogeneity, the type of OVs, and host responsiveness [8]. The specific applications of these different types of OVs in different tumors need to be emphasized. At the same time, facing this dual role, how to seek benefits and avoid harm is a key concern that we need to focus on.

For the aspect of enhancing the beneficial effects of macrophages, gene-regulated OVs demonstrated a strong “re-education” capacity. Various cytokines, chemokines, and immunomodulatory antibodies remodel TAMs and enhance the anti-tumor effects of macrophages. This is one of the modalities with infinite exploits and possibilities that need to be constantly developed and added to.

For the aspect of attenuating the harmful effects of macrophages, we need interventions to avoid unwanted immune responses; after all, intravenous delivery of OVs is a much more promising and ubiquitous mode of administration than local delivery. Direct strategies include the use of immunosuppressive or macrophage-depleting agents for short periods of time to inhibit macrophage capture of OVs. Indirect strategies include the use of genetic modification of viruses to avoid antibody-mediated macrophage clearance and the use of carrier cells or other nanomaterial encapsulation to overcome barriers to intravenous delivery.

We also need to further examine the effect of antigen expression of various viruses on the activation of host immunity, and modification of OVs with extremely low antigen expression without loss of oncolytic biological activity is a major challenge for intravenous delivery. Therefore, we had to elucidate more deeply the interactions among OVs, macrophages, and tumor cells and their mechanisms of action, and utilize the powerful regulatory function of macrophages to synergistically enhance the application of OVs. In future clinical trials of oncolytic virotherapy, it is particularly important to optimize intravenous delivery, avoid unnecessary immune effects, reduce the delivery load of viruses, improve the oncolytic effect of OVs, and enhance the combined therapeutic effect to ultimately achieve a durable and effective treatment outcome.

## Data Availability

Not applicable.

## Abbreviations

OVs: Oncolytic viruses
TME: Tumor microenvironment
TAMs: Tumor-associated macrophages
IFN: Interferon
ICD: Immunogenic cell death
TNF-α: Tumor necrosis factor-α
IL: Interleukin
DCs: Dendritic cells
PAMPs: Pathogen-associated molecular patterns
DAMPs: Damage-associated molecular patterns
TAAs: Tumor-associated antigens
PRRs: Pattern recognition receptors
TLRs: Toll-like receptors
APCs: Antigen-presenting cells
Tregs: Regulatory T cells
TRMs: Tissue-resident macrophages
GM-CSF: Granulocyte–macrophage colony-stimulating factor
CXCL: Chemokines CXC motif ligand
M-CSF: Macrophage colony-stimulating factor
TGF: Transforming growth factor
CSF -1: Colony-stimulating factor 1
HSV: Herpes simplex virus
GBM: Glioblastoma
SIRPα: Signal regulatory protein α
MDMs: Monocyte-derived macrophages
T-VEC: Talimogene laherparepvec
VV: Vaccinia virus
CCL: Chemokine CC motif ligand
CCR: Chemokine CC motif receptor
HAdv-C5: Human species C adenovirus
ICIs: Immune checkpoint inhibitors
